# Shedding light on biology and healthcare—preface to the special issue on Biomedical Optics

**DOI:** 10.1038/s41377-022-00804-w

**Published:** 2022-06-02

**Authors:** Peng Xi, Xunbin Wei, Junle Qu, Valery V. Tuchin

**Affiliations:** 1 Department of Biomedical Engineering, College of Future Technology, Peking University, 100871 Beijing, China; 2 Department of Biomedical Engineering, Peking University, 100081 Beijing, China; 3 Center for Biomedical Optics and Photonics (CBOP) & College of Physics and Optoelectronic Engineering, Key Laboratory of Optoelectronic Devices and Systems of Guangdong Province and Ministry of Education, Shenzhen University, 518060 Shenzhen, China; 4 Saratov State University, 83 Astrakhanskaya str., Saratov, 410012 Russia

**Keywords:** Optics and photonics, Biophotonics

## Abstract

This special issue collects 20 excellent papers, spanning NIR II imaging, high-speed imaging, adaptive wavefront shaping, label-free imaging, ultrasensitive detection, polarization optics, photodynamic therapy, and preclinical applications.

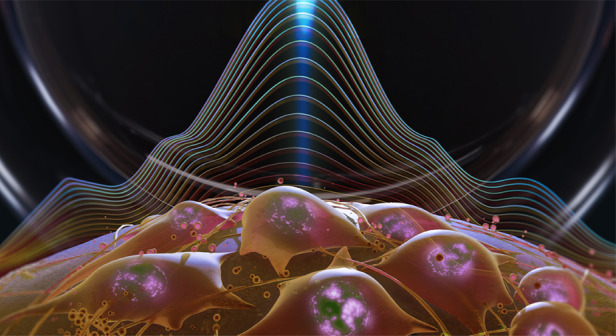

During the past decade, there has been an explosion in the development of novel optical techniques in biomedical research and clinical applications. Immense progress has been made in optical imaging and detection to provide necessary biological information for disease diagnosis and in-depth understanding of biological processes. Moreover, recent advances integrating laser technology with biomarkers have enabled novel diagnostic and treatment approaches for different types of diseases.

The special issue on Biomedical Optics collects 20 excellent papers: 18 original papers and 2 review articles, spanning NIR II imaging, high-speed imaging, adaptive wavefront shaping, label-free imaging, ultrasensitive detection, polarization optics, photodynamic therapy, and preclinical applications.

## NIR II imaging

With 1700 nm optical coherence microscopy, Zhu et al. from the University of California-Davis successfully demonstrated deep label-free imaging, with great image resolution benefitting from the low scattering coefficient at 1700 nm^1^. With this technique, divergent superficial and deep cortical layers were observed in an Alzheimer’s disease (AD) mouse model. This was supported by Feng et al.’s work from Zhejiang University, in which the deep imaging performance of NIR II illumination has been studied extensively^2^. Moreover, Tao et al. from Shanghai Jiaotong University reported that 1070-nm light pulses at 10 Hz can trigger microglial rather than astrocyte responses in AD mice, paving a new avenue for AD treatment^3^.

Taking advantage of the hot-band absorption of indocyanine green (ICG), a dye approved by the US Food and Drug Administration for clinical use, anti-Stokes fluorescence imaging was first demonstrated by a joint research team from China and the US^4^.

## High-speed imaging

To address the missing cone problem in conventional light-field microscopy (LFM), Xiong et al. from Tsinghua University developed mirror-enhanced LFM, which realizes long-term high-speed imaging with isotropic 3D resolution. The technique has been applied to the imaging of various cellular organelle interactions and stable 3D blood cell tracking in zebrafish larvae at volume rates as high as 18 Hz^5^. Zhao et al. from Beihang University presented halftone spatial frequency domain imaging (SFDI), realizing kilohertz high-speed (two orders of magnitude faster than the state-of-the-art technique) label-free imaging by using DMD for modulation of illumination. This approach can be applied to noncontact wide-field imaging and quantification of the optical properties of strongly turbid media^6^.

## Adaptive imaging and wavefront shaping

Digital micromirror devices (DMDs) have played a key role in both display and biological microscopy. In this issue, Abouakil et al. from Aix Marseille University in France have reported two approaches of adaptive imaging of biological surfaces by using DMDs; both approaches yield over 20-fold reduction in light dose^7^. Yang et al. from Beihang University achieved fast wavefront shaping for anti-scattering light focusing with synthetic DMD multipixel encoding^8^. Compared with state-of-the-art DMD-based wavefront shaping, this technique increased the speed of optimization and enhancement of focus by 179- and 16-fold, respectively. DMD has also been applied in halftone SFDI for fast illumination pattern generation^6^.

## Label-free imaging

With 3D volumetric transport optical diffraction tomography and synthetic aperture, Li et al. from Nanjing University of Science and Technology reported a novel label-free microscopy technique, which achieves 200 nm lateral and 500 nm axial resolution, on both cell lines and *C. elegans* imaging^9^. By using Monte-Carlo simulation following with experimental validation, Li et al. from Huazhong University of Science and Technology have demonstrated that the transmissive-detected laser speckle contrast imaging showing superior performance over reflective-detection counterparts for blood flow monitoring in thick tissue, on different samples such as tissue phantom, animal, and human hands^10^.

## Polarization effects

Polarization plays an important role in biomedical imaging. With single-shot illumination, Tang et al. from University of Washington traced the evolution of the polarization state in depth along the Poincare sphere, achieving depth-resolved collagen imaging on both an animal model (rodent heart, ex vivo) and human subjects (face, in vivo) with polarization-sensitive OCT^11^.

In fluorescence microscopy, Guan et al. from Peking University and Tsinghua University reported the combination of lock-in detection with the fluorescence polarization detection, yielding the detection of a universal fluorescence anisotropy of subcellular organelles in live cells, reflecting subtle heterogeneity of the subcellular compartments that was too weak to detect before^12^.

He et al. from Oxford University have contributed a comprehensive review on the application of polarization optics in biomedical and clinical study. Cell and tissue polarimetry related methodologies and applications were covered in the Stokes–Mueller expressions^13^. Recent breakthroughs, development trends, and emerging multimodal techniques are also discussed.

## Ultrasensitive detection

Through plasmonic spectral comb, researchers from Tsinghua University and Jinan University developed a simple optical fiber biosensing platform for ultrasensitive detection of endocrine disruptors, with sensitivity of environmental estrogens detection down to nanogram per liter level^14^.

An international team led by researchers from Shenzhen University (China) and Buffalo University (USA) has proposed a novel fluorescence lifetime imaging assay to study the fine genomic structural alterations such as DNA compaction, replication, and gene expression, based on the lifetime change of DNA fluorescence probes in responding to their local refractive index, and the Förster resonance energy transfer (FRET) process^15^.

## Photodynamic therapy

PDT has been applied as a clinically approved treatment for cancers, despite its limitations, such as relatively low efficiency and the inability to penetrate deep tissue. In this issue, Shramova et al. from the Russian Academy of Sciences developed genetically encoded bioluminescence resonance energy transfer (BRET)-activated photodynamic therapy toward a self-glowing, deep-tissue “photodynamic” therapy without any external light source^16^. With polymer-encapsulated carbonized hemin nanoparticles as photosensitizers, Lin et al. from Shanghai Jiao-tong University demonstrated superior in vitro and in vivo PDT effects through improved ROS generation efficiency, hypoxia relief, and glutathione depletion^17^.

## Preclinical applications

Pshenay-Severin et al. from the Russian Academy of Sciences reported an ultracompact fiber-scanning endoscope platform for multimodal nonlinear endomicroscopy^18^. Based on a powerful, compact four-wave mixing-based fiber laser and a 2.4 mm diameter NIR-dual-waveband corrected endomicroscopic high-numerical-aperture objective, ultrahigh spatiotemporal resolution tissue imaging at 1 fps with submicron spatial resolution has been achieved.

Furthermore, with fluorescence in vivo flow cytometry (IVFC), circulating tumor cells (CTCs) were monitored noninvasively in an orthotopic mouse model of human prostate cancer^19^. Researchers from Shanghai Jiaotong University and Peking University found that CTCs exhibited stochastic bursts over cancer progression. IVFC has also been applied to hematologic malignancy models by Williams et al.^20,21^.

Optical coherence tomography angiography (OCTA) has become an emerging tool for retinal diagnosis. In a review paper, authors from the University of Surrey in the UK listed the key steps for standardizing OCTA imaging of the human retina, analyzed issues and inconsistencies, and proposed minimum standards for imaging protocols, data analysis methods, metrics, reporting of findings, and clinical practice, as well as possible areas that require further investigation^22^.

We hope you enjoy reading these exciting breakthroughs. We thank all the authors and reviewers for their strong support of this special issue. Additionally, we are grateful to the editors of *Light: Science and Applications* for giving us the opportunity to organize this special issue. Last but not least, we express our gratitude to the editorial team of *Light: Science and Applications* for their excellent and professional work.
